# Turner Syndrome and the Risks of Clinical Depression in Adolescence

**DOI:** 10.7759/cureus.19204

**Published:** 2021-11-02

**Authors:** Mayank Gupta, Faiza Zubiar, Nihit Gupta

**Affiliations:** 1 Psychiatry, Lake Erie College of Osteopathic Medicine, Erie, USA; 2 Psychiatry and Behavioral Sciences, Clarion Psychiatric Center, Clarion, USA; 3 Psychiatry, The Trenton Psychiatric Hospital, Trenton, USA; 4 Psychiatry, University of West Virginia, Glen Dale, USA

**Keywords:** antidepressant, pediatric genetics, adolescents, clinical depression, turner syndrome

## Abstract

According to the Center for Disease Control (CDC), 3.2% of children aged 3-17 years have been diagnosed with depression. Many genetic conditions predispose children and adolescents to various mental health problems. Turner syndrome is a common sporadic genetic condition in females with medical issues, developmental delays, and psychiatric comorbidities. There is limited literature about adolescents with a late diagnosis of Turner syndrome and struggles with affective psychopathology. The early recognition and understanding of its unique genetics, neurobiology, and specific clinical manifestations are critical for addressing the needs of these patients.

## Introduction

Turner syndrome (TS), a genetic disorder characterized by the total or partial absence of an X chromosome in females, is the most common sex chromosome abnormality in females affecting one in every 2,000-2,500 live female births [1]. Its association with medical and developmental issues, such as short stature, gonadal dysgenesis, weblike neck, slowed growth, abnormalities in eyes and ears, heart problems, infertility, renal and gastrointestinal problems, skeletal anomalies, and others is well established [2]. Generally, women with TS have average intelligence; however, learning disabilities have been reported in some cases [3]. Verbal IQ is usually higher than performance IQ; studies found an average verbal IQ of 101 compared to an average performance IQ of 89 [4]. Literature suggests structural and functional changes in the brain in cases of TS; however, its association with psychiatric disorders is less known [5,6]. Individuals with TS are at higher risk of being diagnosed with depression, anxiety disorders, mood disorders, autism spectrum disorders, attention deficit hyperactivity disorder, schizophrenia, and psychotic disorders [7]. Social difficulties appear to be an area of vulnerability for young women. Research shows a possible association between age at diagnosis and increased substance use and depressive symptoms. There are few reports of psychiatric manifestations in children and adolescents with TS [8,9].

We present a case report of a 16-year-old adolescent with mosaic TS and psychiatric comorbidities.

## Case presentation

A 16-year-old Asian American female; was admitted to the inpatient unit with depression and suicidal thoughts. The patient reports subsyndromal depressive symptoms for the two years, which have worsened in the last two months. Over the past several months, she started self-mutilating by cutting her arms and legs with a razor. She also had suicidal ideations with a plan to stab herself with a knife or get hit by a car. The precipitating event was being accused of cheating on a school test, and the fear of being expelled. The mother reported the patient was having increased sleep, decreased motivation, social isolation, and dropping school grades. Besides school stress, the patient reported conflict with her parents about the cultural differences. The patient stopped talking to her parents, siblings, and her teachers. She stopped doing homework or studying for tests; her grades dropped from A’s & B’s to C’s & D’s. She did admit to suicidal ideation for months, without a plan. The patient was born and raised in Asia until she was seven when her parents moved to the United States. Her parents expected her to follow their own culture and religion, which had posed many arguments in the household. The patient’s father worked at the same school, and she struggled between rigid rules of her parents’ traditions, and culture and finding a balance to maintain her own cultural identity.

When the patient was in eighth grade, she was diagnosed with TS but was never disclosed to her. She was born after a full-term pregnancy with a caesarian section delivery and met all her milestones. The patient reported experiencing bullying since the fifth grade for being short. She started taking growth hormones in eighth grade but stopped one month ago when she became severely depressed. She continued to elaborate that she had low self-esteem because she felt that something was wrong with her. She described that her mother would occasionally meet with the doctor while she waited outside the room. She was told to have needed growth hormones because she was not growing appropriately. She was in 10th grade and had never repeated a grade. She reported that she was in the process of getting an Individual Educational Plan (IEP) evaluation as it took her extra time to finish papers, projects, and tests when compared to other students in her class. She denied any use of tobacco, alcohol, or illicit substances. She denied any history of physical or sexual abuse. She has been prescribed Sertraline 25mg after the consent of the mother and ascent of the patient. After one week, the Sertraline was titrated to 50mg and then to 75mg after the patient showed a minimal response. After week 2, the patient reported that she felt that the medication was slightly helpful, and she was more motivated about her future. However, there would be many times when she felt worthless. Sertraline was again increased to 100mg daily. At first, the patient had social difficulties with peers but reluctantly started to participate in group therapy. She reported having learned about herself in groups. After a few family sessions, the patient continued to feel rejected, misunderstood, and not ready to accept feedback. The day after this family session, the patient took a downturn and was punching walls and verbally aggressive. She also expressed that living with a family friend out of the United States might be an option as she did not want to return home. She agreed that listening to music and blogging on the computer were her coping skills at home and could add writing, drawing, and stretching to her list of other possibilities. Aripiprazole 2mg was added to her medication regime to augment the action of Sertraline. After a week on this combination regimen, she showed more improvement with her depressive symptoms. In the next family session, she agreed to step down to the partial hospital and continue to work on the family issues with her therapist. She was discharged back home in the care of her mother.

## Discussion

Most previous studies have examined psychiatric disorders associated with TS in adults, whereas fewer studies have focused on depression in children and adolescents with TS and depression. A recent study has linked delay in the diagnosis of TS after the age of 13 with the risk of depression and substance use disorders [10]. TS is a sporadic and common condition not affiliated with advanced maternal age. Studies using Xg blood grouping, restriction-fragment length polymorphism, and single nucleotide polymorphism have shown that the missing X chromosome is typically paternal in origin, constituting approximately 75% of live births [11]. If a cell is determined to be 45, X in amniotic fluid or chorionic villus sampling, fluorescence in situ hybridization (FISH) testing with X and Y centromere probes is the next step to determine the level of mosaicism [12]. Parental origin of the intact X chromosome may also affect the phenotype of TS. In a study women with maternal X chromosome are active (45, Xm) performed poorly in areas of social cognition and adjustment than women whose paternal X chromosome is active (45, Xp). Skuse et al. found that verbal IQ was significantly lower in women with 45, Xm as compared to 45, Xp [13]. The level of mosaicism may help determine the effects on cognition. There is a myriad range of phenotypes due to the genetic mutations involving the X chromosome. In TS the haploinsufficiency of multiple genes of the X chromosome or loss of the Y chromosome may affect the embryologic development, stature, and gonadal functions. Specifically, short stature likely results from the deletion of one allele of SHOX (short stature homeobox gene), a transcription factor on Xp22. Also, it has been shown that patients without TS but that have idiopathic short stature may also harbor mutations in SHOX that affect this phenotype [14]. Other X chromosome abnormalities, such as deletions affecting Xp11 may result in ovarian failure or failure in menstrual functions. There may also be an increased risk of Hashimoto thyroiditis and hypothyroidism if an isochromosome of X is present.

With the many phenotypic abnormalities associated with the aberrant X chromosome, it is plausible that mental illness in TS patients may also originate from errors in the X chromosome. Thus, genotypic influences may potentially provide important insights into the study of social behavior and emotional difficulties faced by women with TS. There are many studies of women with TS that highlight the anatomical and functional differences in the structure of the brain. Specifically, changes in the prefrontal cortices, superior temporal gyrus, hippocampal formation, amygdala, and temporal lobes have been shown in women with TS [15,16]. Interestingly, amygdala hypermetabolism and cortisol release are linked to depression [17]. Studies have suggested that dysfunction of the prefrontal cortex, particularly concerning its role in modulating limbic activity, could be seen in clinical depression [18]. The hippocampus impacts how the brain processes memory and learning, as well as the ability to understand spatial contexts. It has been seen in depression subjects the hippocampus region is smaller in size than one without any symptoms of depression [19]. Therefore, the changes in brain morphology associated with the missing X chromosome seen in TS could be linked to depression. There are only a few studies that have reported cognitive deficits in children with TS. However, many studies have found high rates of social anxiety disorder with TS and also have higher amygdala volumes [20].

## Conclusions

It is imperative to assess the adolescents with TS for affective dysregulation to provide early interventions. Therefore, screening for developmental disorders and psychiatric symptoms is critical to address the co-occurring mental health conditions. Given the complex clinical presentations of affective psychopathology in TS, there may be a role of pharmacogenetic testing in select refractory cases. The multidisciplinary clinical team with parents and special educators may work concurrently to address the needs of these youths.
